# Risk Assessment for Self Reported Obstructive Sleep Apnea and Excessive Daytime Sleepiness in a Greek Nursing Staff Population

**DOI:** 10.3390/medicina55080468

**Published:** 2019-08-12

**Authors:** Alexia Alexandropoulou, Georgios D. Vavougios, Chrissi Hatzoglou, Konstantinos I. Gourgoulianis, Sotirios G. Zarogiannis

**Affiliations:** 1 Department of Physiology, Faculty of Medicine, University of Thessaly, BIOPOLIS, 41500 Larissa, Greece; 2 Department of Neurology, Athens Naval Hospital, 11521 Athens, Greece; 3 Department of Respiratory Medicine, Faculty of Medicine, University of Thessaly, BIOPOLIS, 41500 Larissa, Greece

**Keywords:** Berlin Questionnaire, Epworth Sleepiness Scale, nursing staff, Obstructive Sleep Apnea Syndrome, risk assessment

## Abstract

*Background and objectives*: The risk assessment of Obstructive Sleep Apnea (OSA) and Excessive Daytime Sleepiness (EDS) in specific occupational populations is important due to its association with morbidity. The aim of the present study was to identify the risk of OSA development and EDS in a Greek nursing staff population. *Materials and Methods*: In this cross-sectional study a total of 444 nurses, 56 males (age = 42.91 ± 5.76 years/BMI = 27.17 ± 4.32) and 388 females (age = 41.41 ± 5.92 years/BMI = 25.08 ± 4.43) working in a Greek secondary and tertiary hospital participated during the period from 18 January 2015 to 10 February 2015. The participants completed the Berlin Questionnaire (BQ), concerning the risk for OSA and the Epworth Sleepiness Scale (ESS), concerning the EDS. The work and lifestyle habits of the participants were correlated with the results of the questionnaires. *Results*: According to the BQ results 20.5% (*n* = 91) of the nursing staff was at high risk for OSA. Increased daytime sleepiness affected 27.7% (*n* = 123) of the nurses according to ESS results. Nurses at risk for Obstructive Sleep Apnea Syndrome (OSAS), positive for both BQ and ESS, were 7.66% (*n* = 34). Out of the nurses that participated 77% (*n* = 342) were working in shifts status and had significant meal instability (breakfast *p* < 0.0001, lunch *p* < 0.0001, dinner *p* = 0.0008). *Conclusions*: The population at high risk for OSA and EDS in the nursing staff was found to be 20% and 28% respectively. High risk for OSAS was detected in 7.66% of the participants. The high risk for OSA and EDS was the same irrespective of working in shift status. In specific, nursing population age was an independent predictor for high risk for OSA and skipping lunch an independent predictor of daytime sleepiness.

## 1. Introduction

The World Health Organization (WHO) indicates that Obstructive Sleep Apnea Syndrome (OSAS) is a preventable lung disease [1]. Most patients with this syndrome exhibit no detectable respiratory dysfunction when awake while OSAS appears in all age groups. However, in the adult population the incidence of this syndrome increases with age and is clearly linked with excessive daytime sleepiness (EDS) [1,2,3].

The prevalence of this syndrome is probably higher than the one presumed due to underdiagnosis. Thus, OSAS constitutes an important public health issue [4,5,6,7]. It is estimated that 26% of the worldwide adult population is at high risk for developing the syndrome [4]. Epidemiological studies indicate that the exact determination of OSAS prevalence is difficult due to different methodological approaches [3,8,9]. Studies from USA, Australia, India, China and Korea report that the prevalence in the general adult population spans from 3 to 7% in men and 2 to 5% in women to more than 49% depending on age and gender [5,6,7,9,10,11,12,13,14,15]. This reported non-uniformity of the prevalence in 4 different continents strengthens the notion that the disease is common but the prevalence in the community needs to be studied more rigorously in order to avoid underdiagnosis [4,8].

The investigation of OSAS in the context of specific occupations is of high interest given that its occurrence may be associated with working conditions that do not only induce the disease but also affect the job performance and overall health [16,17,18]. A study on American and Canadian police officers showed that this specific population has at least one sleep disorder. Moreover, one third of the study population, suffered from OSAS (33.6%) [19]. Another study in young male Korean soldiers using the Berlin Questionnaire (BQ) reported a prevalence of 8.1% of OSA [20]. In a similar study conducted in the staff of an Iranian hospital again with the BQ tool, it was found that 6.9% were at high risk for OSAS. Finally, a study in 21 nurses, showed that according to the BQ, 24% were at high risk for OSA, but subsequent polysomnography revealed that 43% of them were diagnosed with OSAS [16].

No studies exist in the Greek population regarding the risk assessment of self-reported OSA and EDS in specific occupational groups. Given that the nursing staff work several times in shift status, which has been implicated in the induction of OSAS [21,22], we hypothesized that this population would be under risk for developing OSA and EDS, due to their sleep fragmentation. Thus, the aim of the present study was to identify the nursing population at high risk for OSA and EDS in a secondary and tertiary hospital in Greece.

## 2. Materials and Methods

### 2.1. Study Population

The study population consisted of 444 nurses working in the University Hospital and the General Hospital of Larissa during the period 18 January 2015 to 10 February 2015 who volunteered to participate in the study. In total, 530 questionnaires were distributed by the primary author in personal communication with the potential participants. The potential participants were given a week to complete the questionnaires and were asked to put them in an un-named envelope and hand them to a designated administrative officer of each hospital sector (Medical, Surgical and Intensive Care) for collection by the primary author. Out of the 530 questionnaires 449 were returned to the primary author. Out of the 449 questionnaires, 5 were not fully completed and were thus excluded from the study, leading to a final number of 444 questionnaires included in the study. The study involved 56 male (12.6%) and 388 female (87.4%) nurses, regardless of educational level and work experience, from all nursing departments. All participants provided demographic information such as gender, age, height, weight, smoking habits, skipping meals and whether they worked under night shifts. The Ethics Committee of the University Hospital of Larissa approved the research protocol (Protocol number: 1/14-1-2015).

### 2.2. OSAS and EDS Assessment Tools

In order to assess the risk of OSAS the Greek version of the Berlin Questionnaire (BQ) was used [23]. The BQ contains 10 questions that are divided in 3 categories. In the first category the questions aim at identifying the self-reported snoring behavior along with witnessed apneas during sleep by the partner. The second category assesses self-reported fatigue after sleep and the third assesses the presence of obesity or history of hypertension. If two of the categories of the BQ are positive, then the participant is assigned as being at high risk for OSAS.

In order to assess the excessive daytime sleepiness, the Greek version of the Epworth Sleepiness Scale (ESS) was used [24]. ESS aims at the quantification of daytime sleepiness though a set of self-reported incidents of dozing in eight different setting during the day and the scoring spans from 0 to 24. A participant in high risk for daytime sleepiness has a score of 10 or higher.

### 2.3. Statistical Analysis

Statistical analysis was performed by EpiInfo v. 7.0 (CDC, Atlanta, GA, USA), the SPSS 24.0 Software (IBM Corporation, New York, NY, USA) and GraphPad Prism v. 8.1 (San Diego, CA, USA). Fisher’s exact was used to assess differences among proportions. The Mann Whitney test was used to assess differences between two groups. Multivariate logistic regression was performed as in other similar studies [25]. The Forward Conditional Logistic Regression Model was used to perform multivariate analyses of the effect of univariate predictors on the likelihood of belonging to the high OSA probability (based on BQ) or high daytime sleepiness (Based on Epworth Scale) groups, while controlling for potential confounders. Values are expressed as mean ± S.D. A *p* value of less than 0.05 was deemed significant.

## 3. Results

### 3.1. Study Population

Out of the 530 questionnaires that were distributed, 444 were completed and collected, providing a responsiveness rate of 83.8%. The demographics of the study population along with lifestyle habits are shown in Table 1.

### 3.2. BQ and ESS Questionnaire Results

**Figure 1:**
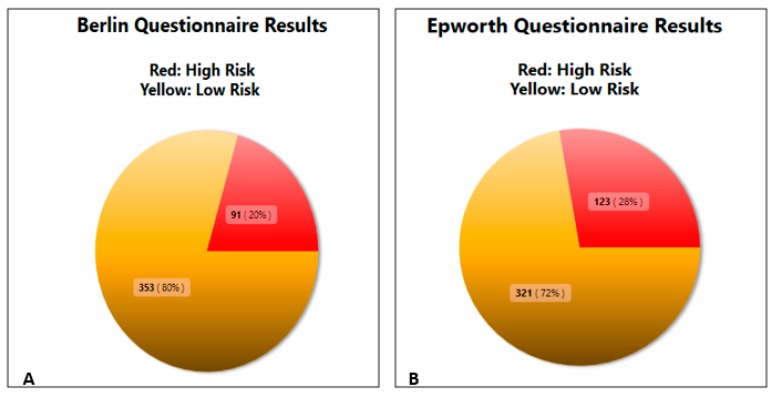
(**A**) Results of the Berlin Questionnaire (BQ) showing 20% of the participants at high risk for Obstructive Sleep Apnea (OSA). (**B**) Results of the Epworth Sleepiness Scale (ESS) showing 28% of the participants at high risk for Excessive Daytime Sleepiness (EDS).

The results of the BQ questionnaire showed that 20% (*n* = 91) of the participants were found to be at high risk for OSAS as opposed to 80% (353) that were found to be at low risk for OSAS (Figure 1A). With regards to the ESS questionnaire 28% (*n* = 123) of the participants were found to be at high risk for EDS as opposed to the 72% (*n* = 321) that were found at low risk (Figure 1B).

More importantly a fraction of these two groups mounting to 8% (*n* = 34) were found to be at concomitant high risk for both OSA and EDS, thus at OSAS risk.

There were no differences in the proportions of male and female nurses that were positive in BQ (*p* > 0.99) or ESS (*p* = 0.75). Working under night shift status did not result in a higher proportion of nurses to test positive in BQ (*p* = 0.69), but resulted in a higher proportion of nurses testing positive in ESS (*p* = 0.005).

**Figure 2:**
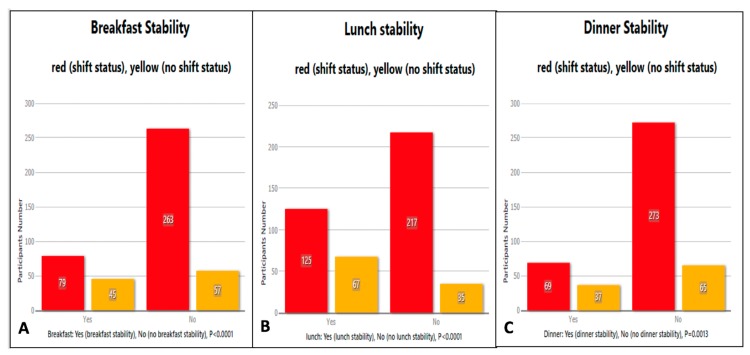
Significant meal instability (skipping of a meal) in the nursing staff working on shift work status regarding (**A**) breakfast, (**B**) lunch and (**C**) dinner.

Another significant finding of our study was that the nursing staff working on shift work status reported skipping meals significantly more than the nurses not under shift status that had a greater stability in maintaining the three main meals of the day as shown in Figure 2.

The Forward Conditional Binary Logistic Regression model was subsequently used in order to determine the effects of age, sex, alcohol consumption, education level, smoking status and breakfast/lunch/dinner skipping on the likelihood of belonging to the (a) high OSA risk and (b) high daytime sleepiness groups. Age was the single independent predictor of belonging to the high risk OSA group [OR: 0.959 (95% CI: 0.922–0.998), *p*-value = 0.038], whereas lunch skipping [OR: 1.631, (95% CI: 1.060–2.509), *p* = 0.026] independently predicted higher daytime sleepiness.

## 4. Discussion

The aim of the present investigation was the identification of the risk for OSA and EDS in the nursing population of a secondary and tertiary hospital in Greece using the standard questionnaires. Our results showed that the nursing population at high risk for OSA was 20%, while that of EDS was 28%. The fraction of the study population that was at high risk for both, and therefore at high OSAS risk, was 8%. Moreover, according to our results, working in shift status did not directly affect the risk for OSA. However, it significantly worsened EDS in the nursing population. It has to be taken into account that the sensitivity and specificity of BQ for OSA diagnosis in the Greek population has been shown to be 76% and 40%, respectively, while the Greek version of ESS had also proved a useful tool for the identification of EDS in Greece [23,24]. In the studied population after multivariate logistic regression it was shown that age was a significant predictor of high risk for OSA, while skipping lunch was an independent prognosticator for high EDS risk. Aging is a known risk factor for OSA development so our result is in line with the literature [26]. As far as lunch skipping is concerned, a study that focused on the daytime sleepiness of subjects during the Ramadan intermittent fasting showed that this intentional prolonged daytime abstinence from food intake, induced an increase in the objective and subjective daytime sleepiness of the subjects [27]. Thus, based on the ESS scores of the participants of our study, we are in agreement with the notion that daytime food abstinence increases the propensity for subjective daytime sleepiness.

There is lack of studies on the risk assessment for OSA and EDS in nursing populations in Greece, so our results cannot be compared to the published literature. However, a similar study performed in the USA in nurses working in shift status using the BQ, showed that 24% of participants were at risk for OSA [16]. Although our study had a sample size nearly 20-fold bigger the above-mentioned results are comparable to ours that showed that 20% of the population was at high risk for OSA. On the other hand, in the study of Geiger-Brown et al., after polysomnography 43% of the participating nurses were diagnosed with sleep-disordered breathing, therefore if we extrapolate these findings to our study, we should expect a significantly higher number of nurses with OSAS in our sample. This was a limitation of our study but is the topic of a new investigation currently underway. A previous Greek study has shown that in subjects that underwent polysomnography, OSAS was diagnosed five times more in men than women, nevertheless using the BQ we did not detect such a difference between genders [28].

Data stemming from other occupational groups have reported comparable results. A cross-sectional and prospective cohort study in police officers in North America that involved a 10-fold greater population (*n* = 4957) than ours indicated that 40.1% of the police staff had at least one sleep disorder [29]. The most important of these disorders that was observed in 33.6% was OSAS. A total of 28.5% of the police staff also had EDS and the significant possibility to sleep during driving (once a month).

The failure of sleep replenishment during the day and the abnormality of melatonin levels affects the daily fatigue and reduces the quality of life [18]. In our study, 28% of the nurses were found to have EDS according to the ESS results. This finding is alerting under the rationale that sleep deprivation can lead to reduced concentration and productivity, and also in increased traffic and occupational accidents and injuries, as well as chronic diseases (cardiovascular and metabolic) and reduced quality of life [16,17,18,21,22]. Although no relevant data exists in the literature regarding Greece in order to be able to compare our findings, we are in good agreement with studies performed in nurses in New Zealand (that reported 33.75% of positive ESS) and Sweden (that reported 32.5% of positive ESS with a cut-off of 9 that was different from the one in our study that was 10) [30,31]. Our results were higher than a recent study performed in China that reported 16.1% positive ESS but the cut-off the authors used was 14, therefore these results are not directly comparable with the current study [32]. Another important finding of our study was that the nursing population working in shift status had significant instability in all three main meals of the day as determined by the self-report of participants of meal skipping. It has been reported that the instability of meals induces increases in body weight and thus BMI [18]. Obesity is a known risk factor for OSAS and furthermore a specific type of sleep apnea is observed in this population, the Obesity Hypoventilation Syndrome (OHS) [3,16]. Indeed, in our study the participants that were found to be in the high risk for OSA based on BQ results were significantly heavier that the ones in the low-risk group. Moreover, shift status has been implicated in the induction of OSAS [21,22]. Shift-work disrupts the expression of circadian genes and sleep patterns, deregulates metabolic processes and can cause sleep apnea and several disorders linked to OSAS like cardiovascular disorders and obesity [33].

There were some limitations in the current study. The population of our study was young since most nurses were in their early forties predominantly. Additionally, our sample comprised predominantly of females and this may have diluted the significance of our results. Finally, all participants were originating from a single geographic area and thus further multicenter studies are needed.

## 5. Conclusions

In conclusion, we found that the risk for OSA and EDS in a nursing population of a secondary and tertiary hospital in Greece was 20% and 28% respectively. At high risk for OSAS were 8% of the participants (positive BQ and ESS simultaneously). Moreover, we found that nurses that work under night shift status had significant meal instability, which is a risk factor of obesity, which is in turn linked to OSAS development. However, we detected no differences in OSAS risk between these two groups of the population assessed. Further study of the population under high risk for OSAS of this study involving polysomnography assessment is needed.

## Figures and Tables

**Table 1:** Characteristics of the participants in the study.

	Males	Females	*p* Value
Gender (%)	56 (12.6%)	388 (87.4%)	-
Age (years)	42.91 ± 5.76	41.41 ± 5.92	0.047
Height (m)	1.78 ± 0.06	1.65 ± 0.05	<0.001
Weight (kg)	86.05 ± 14.30	67.91 ± 12.03	<0.001
BMI	27.17 ± 4.32	25.08 ± 4.43	<0.001
Smokers—Yes(#)	29 (51.79%)	171 (44.07%)	0.39
Pack Years (#)	14.61 ± 11.31	15.78 ± 11.18	0.59
Alcohol Consumption—Yes (%)	40 (70.42%)	192 (49.48%)	0.004
Working on Night Shifts—Yes (%)	44 (78.57%)	287 (73.97%)	0.52
Night Shifts per month (#)	4.43 ± 1.87	6.25 ± 2.25	<0.001

## References

[1] Veale D. (2006). Chronic respiratory care and rehabilitation in France. Chron. Respir. Dis..

[2] Berry R.B., Budhiraja R., Gottlieb D.J., Gozal D., Iber C., Kapur V.K., Marcus C.L., Mehra R., Parthasarathy S., Quan S.F. (2012). Rules for scoring respiratory events in sleep: Update of the 2007 AASM manual for the scoring of sleep and associated events. J. Clin. Sleep Med..

[3] Jennum P., Riha R.L. (2009). Epidemiology of sleep apnoea/hypopnoea syndrome and sleep-disordered breathing. Eur. Respir. J..

[4] Carlucci M., Smith M., Corbridge S.J. (2013). Poor sleep, hazardous breathing: an overview of obstructive sleep apnea. Nurse Pract..

[5] Peppard P.E., Young T., Barnet J.H., Palta M., Hagen E.W., Hla K.M. (2013). Increased prevalence of sleep-disordered breathing in adults. Am. J. Epidemiol..

[6] Heinzer R., Vat S., Marques-Vidal P., Marti-Soler H., Andries D., Tobback N., Mooser V., Preisig M., Malhotra A., Waeber G. (2015). Prevalence of sleep-disordered breathing in the general population: The HypnoLaus study. Lancet Respir. Med..

[7] Simpson L., Hillman D.R., Cooper M.N., Ward K.L., Hunter M., Cullen S., James A., Palmer L.J., Mukherjee S., Eastwood P. (2013). High prevalence of undiagnosed obstructive sleep apnoea in the general population and methods for screening for representative controls. Sleep Breath..

[8] Punjabi N.M. (2008). The epidemiology of adult obstructive sleep apnea. Proc. Am. Thorac. Soc..

[9] Mirrakhimov A.E., Sooronbaev T., Mirrakhimov E.M. (2013). Prevalence of obstructive sleep apnea in Asian adults: a systematic review of the literature. BMC Pulm. Med..

[10] Sharma S.K., Ahluwalia G. (2010). Epidemiology of adult obstructive sleep apnoea syndrome in India. Indian J. Med. Res..

[11] Mahboub B., Afzal S., Alhariri H., Alzaabi A., Vats M., Soans A. (2013). Prevalence of symptoms and risk of sleep apnea in Dubai, UAE. Int. J. Gen. Med..

[12] BaHammam A.S., Alrajeh M.S., Al-Jahdali H.H., BinSaeed A.A. (2008). Prevalence of symptoms and risk of sleep apnea in middle-aged Saudi males in primary care. Saudi Med. J..

[13] Kang K., Seo J.G., Seo S.H., Park K.S., Lee H.W. (2014). Prevalence and related factors for high-risk of obstructive sleep apnea in a large Korean population: Results of a questionnaire-based study. J. Clin. Neurol..

[14] Tufik S., Santos-Silva R., Taddei J.A., Bittencourt L.R.A. (2010). Obstructive Sleep Apnea Syndrome in the Sao Paulo Epidemiologic Sleep Study. Sleep Med..

[15] Akkoyunlu M.E., Altin R., Kart L., Atalay F., Örnek T., Bayram M., Tor M. (2013). Investigation of obstructive sleep apnoea syndrome prevalence among long-distance drivers from Zonguldak, Turkey. Multidiscip. Respir. Med..

[16] Geiger-Brown J., Rogers V.E., Han K., Trinkoff A., Bausell R.B., Scharf S.M. (2013). Occupational screening for sleep disorders in 12-h shift nurses using the Berlin Questionnaire. Sleep Breath..

[17] Simpson G. (2011). Circadian rhythm sleep disorders. J. R. Coll. Physicians Edinb..

[18] Rajaratnam S.M.W., Howard M.E., Grunstein R.R. (2013). Sleep loss and circadian disruption in shift work: health burden and management. Med. J. Aust..

[19] Ko H.S., Kim M.Y., Kim Y.H., Lee J., Park Y.G., Moon H.B., Kil K.C., Lee G., Kim S.J., Shin J.C. (2013). Obstructive sleep apnea screening and perinatal outcomes in Korean pregnant women. Arch. Gynecol. Obstet..

[20] Lee Y.C., Eun Y.G., Shin S.Y., Kim S.W. (2013). Prevalence of snoring and high risk of obstructive sleep apnea syndrome in young male soldiers in Korea. J. Korean Med. Sci..

[21] Laudencka A., Klawe J.J., Tafil-Klawe M., Zlomanczuk P. (2007). Does night-shift work induce apnea events in osbtructive sleep apnea patients?. J. Physiol. Pharmacol..

[22] Paciorek M., Korczynski P., Bielicki P., Byśkiniewicz K., Zieliński J., Chazan R. (2011). Obstructive sleep apnea in shift workers. Sleep Med..

[23] Bouloukaki I., Komninos I.D., Mermigkis C., Micheli K., Komninou M., Moniaki V., Mauroudi E., Siafakas N.M., Schiza S.E. (2013). Translation and validation of Berlin questionnaire in primary health care in Greece. BMC Pulm. Med..

[24] Tsara V., Serasli E., Amfilochiou A., Constantinidis T., Christaki P. (2004). Greek version of the Epworth Sleepiness Scale. Sleep Breath..

[25] Romandini M., Gioco G., Perfetti G., Deli G., Staderini E., Laforì A. (2017). The association between periodontitis and sleep duration. J. Clin. Periodontol..

[26] Gaspar L.S., Alvaro A.R., Moita J., Cavadas C. (2017). Obstructive sleep apnea and hallmarks of aging. Trends Mol. Med..

[27] Roky R., Chapotot F., Benchekroun M.T., Benaji B., Hakkou F., Elkhalifi H., Buguet A. (2003). Daytime sleepiness during Ramadan intermittent fasting: polysomnographic and quantitative waking EEG study. J. Sleep Res..

[28] Vagiakis E., Kapsimalis F., Lagogianni I., Perraki H., Minaritzoglou A., Alexandropoulou K., Roussos C., Kryger M. (2006). Gender differences on polysomnographic findings in Greek subjects with obstructive sleep apnea syndrome. Sleep Med..

[29] Barger L.K., Lockley S.W., Shea S.A., Wang W., Landrigan C.P., O’Brien C.S., Qadri S., Sullivan J.P., Cade B.E., Epstein L.J. (2013). Sleep disorders, health and safety in Police Officers. Jama.

[30] Gander P., OKeeffe K., Santos-Fernandez E., Annette H., Leonie W., Jinny W. (2019). Fatigue and nurses’ work patterns: An online questionnaire survey. Int. J. Nurs. Stud..

[31] Brown J.G., Wieroney M., Blair L., Zhu S., Warren J., Scharf S.M., Hinds P.S. (2014). Measuring subjective sleepiness at work in hospital nurses: Validation of a modified delivery format of the Karolinska Sleepiness Scale. Sleep Breath..

[32] Chen L., Luo C., Liu S., Chen W., Liu Y., Li Y., Du Y., Zou H., Pan J. (2019). Excessive daytime sleepiness in general hospital nurses: prevalence, correlates, and its association with adverse events. Sleep Breath..

[33] Khan S., Duan P., Yao L., Hou H. (2018). Shiftwork-mediated disruptions of circadian rhythms and sleep homeostasis cause serious health problems. Int. J. Genomics.

